# Validity and reliability of knowledge, attitude, and practice regarding exercise and exergames experiences questionnaire among high school students

**DOI:** 10.1186/s12889-022-14147-z

**Published:** 2022-09-14

**Authors:** Rwayda Abdulkader Mohamed, Nur Arzuar Abdul Rahim, Siti Mardhiana Mohamad, Hazwani Ahmad Yusof

**Affiliations:** 1 Department of Community Health, Advanced Medical and Dental Institute, Universiti Sains Malaysia, 13200 Kepala Batas, Pulau Pinang, Malaysia; 2 Department of Clinical Medicine, Advanced Medical and Dental Institute, Universiti Sains Malaysia, 13200 Kepala Batas, Pulau Pinang, Malaysia

**Keywords:** Adolescent, Physical activity, Games, Technology

## Abstract

An instrument to measure the Knowledge, Attitude, and Practice (KAP) related to exercise and exergames experiences among high school students is important to identify the KAP gap that may facilitate the health intervention to boost the immunity of the young population. Thus, our study aims to develop and validate a questionnaire assessing KAP regarding exercise and exergames experiences among high school students. A cross-sectional study was conducted among 188 high school students in Malaysia. This research questionnaire was adapted from a previous study and modified with an additional section to assess high school students’ exergames experiences. Content validity, construct validity, and reliability was verified using different tests. Only one item (slimming tea and other drugs could be used in place of exercise to achieve the same effects) did not reach the required level of content validity index in terms of their relevance, clarity, simplicity, and ambiguity. Therefore, this item was removed from the instrument. For factor analysis, three questions were removed from the instrument due to those questions having a factor loading lower than 0.4. The Intraclass Correlation Coefficient values for the test–retest reliability after an interval of two weeks for all questions were > 0.7. Kappa coefficient ranges between 0.67-0.96. The SME value ranged from 0.34 to 1.47. Repeated measured ANOVA did not reveal any significant differences between the test and re-test. This questionnaire has strong validity and reliability that can be used to measure high school student’s knowledge, attitude, and practice about exercise and their exergames experiences.

**Supplementary Information:**

The online version contains supplementary material available at 10.1186/s12889-022-14147-z.

## Introduction

Exercise is a subset of physical activity that is defined as any bodily movement produced by skeletal muscles that result in energy expenditure which increases body calorie output and also heart rate to burn more calories in the body and maintain physical fitness [1]. Exercise is known as planned, structured, and repetitive physical activity [2]. Several health-based organizations recommend children do moderate to vigorous physical activity daily for 60 min [3]. The latest reports demonstrate that 78% of teenagers do not accumulate 60 min or more of physical activity that should include exercise per day [3]. According to the theory of planned behavior, the significance of exercise knowledge to physical activity engagement lies in its relationship with attitude [4]. Specifically, the knowledge component influences the attitudes of an individual’s intention to participate in the exercise. Knowing the health benefits of exercise is the basis for participating in regular exercise activity [5]. Hence, developing the knowledge of exercise and its relationship with health and fitness might be the first stage to the foundation of a positive attitude towards exercise [6–8].

It has been reported that exercise can be the key to protecting, controlling, and decreasing body weight [9]. Physical activity or exercise engagement levels in kids and teenagers have declined dramatically over the last two decades, according to studies, there are a few young people who complied with physical activity or exercise recommendations [10–13]. Therefore, there is a need to determine several efficient strategies to increase the physical activity or exercise engagement level in young people. Given that today’s youth population love to play video game active video games or also known as exergames may provide an alternative way to increase the physical activity level or exercise engagement in our youth’s increasingly technology-driven society [14–17]. Penko and Barkley [18] establish that children and adults incline toward playing Nintendo Wii boxing (not with standing, being all the more physiologically requesting) rather than a more conventional form of exercise (leisurely treadmill walking) and an inactive video game.

A Knowledge, Attitude, and Knowledge (KAP) survey is a quantitative approach with pre-determined questions and standardized questionnaire formats that gives access to both quantitative and qualitative data [19]. The KAP survey is a representative study of a given population that aims to identify what is known, believed, and done regarding a specific topic. They are able to recognize information that is well known and attitudes that are widely held about particular themes. They can, to a certain extent, detect factors influencing behaviour and the causes of people's attitudes, and suggest an intervention strategy based on this information [20].

There are a few versions of KAP on exercise survey that have been established [21–23]. KAP on exercise survey may aid in a situation analysis by identifying exercise priorities by assisting in identifying the current knowledge, attitudes, and practices relative to exercise. Most of the surveys are focusing on adult, elderly, or clinical populations, and limited studies with the high school age populations and exergames. Fabunmi et al. [24] have assessed KAP in physical exercise among Nigerian school students but the survey did not include questions related to exergames. The questionnaire has simple items which can be suitable for this population. The development and validation of the new KAP questionnaire related to exercise and exergames specifically in high school students may provide more evidence regarding KAP among the high school age population for a better understanding of physical activity or exercise-related issues among children. In addition, assessing the exergames experiences of high school students may provide us a preliminary result regarding the feasibility of exergames as innovative tools for individual health.

Therefore, our study aims to develop and validate a questionnaire assessing the KAP regarding exercise and exergames experiences among high school students. The version of the developed and validated KAP questionnaire produced in this study may yield beneficial information for further physical activity-related research among the school-age population.

## Methods

A cross-sectional study was carried out among high school students who met our inclusion criteria: Aged between 13 to 17 years old, high school students in Malaysia, and those able to understand English. Because the questionnaire was written in English, the high school pupils must be able to comprehend it. Given that English is one of the official languages of Malaysia and English is widely spoken in Malaysia. Thus, the questionnaire was written in English. The study population of this study is high school students. High school student in Malaysia is usually aged range between 13–17 years old. Hence, most of our study respondents were of this age. A total of 188 high school students from 42 schools in urban areas of Malaysia participated in this study. The selection of school in urban areas is due to English language proficiency that most prominent among urban students in Malaysia compared to rural students.

The selection of schools in urban areas across Malaysia were randomly chosen by the researchers as long as it is located in urban areas. The questionnaires were distributed to potential respondents by sharing the questionnaire link with the schools’ principals and we requested to share the link within the student’s WhatsApp group. Due to the low response rate of our online questionnaire. So, the questionnaire was shared with many schools to achieve an adequate sample size. The participants and their guardians signed an informed consent form. We obtained approval from the ministry of education Malaysia and The Human Research Ethics Committee of University Sains Malaysia under reference number [USM/JEPeM /21050380].

### Instrument

The questionnaire of this research was adapted from Fabunmi et al. [24] that was modified with an additional section to assess exergames experiences. The questionnaire consists of 39 questions. Part 1 contains five questions about sociodemographic data. Part 2 contains 10 questions regarding knowledge about exercise. Part 3 contains 10 questions about attitudes toward exercise, and Part 4 has nine questions regarding participation in exercise. Part 5 contains five questions regarding exergames’ experiences. The respondents were required to answer the questionnaire based on a three-point Likert scale (agree, neutral, disagree) for the knowledge domain, a five-point scale (strongly agree, agree, neither agree nor disagree, disagree, strongly disagree) for the attitude domain, (yes, no, open answer) for participation domain, and (yes, no) for experiences domain.

### Content validity of the questionnaire

Content validity measures how well items correspond to or reflect a specific domain and are measured using quantitative techniques [25, 26]. When the instrument measure is designed to be measured in terms of relevance and representativeness, this process is known as content validity [27]. Validity is defined as the extent to which any instrument measures what it is intended to [26]. For this reason, the development of the experience’s domain went through multiple iterations to ensure the survey was clearly worded, well defined, and covered the topics important to the exergames experiences. All questions were assessed by six experts as follows: Two exercise scientists with experience in exercise research among adolescents, two public health specialists with experience in the health status of children and adolescents, one pediatrician with experience in the well-being of adolescent assessment, physical education teacher with experience in dealing with high school students about physical education activity at school. The experts have been given a scale from 1 to 4 to assess the questions based on relevance, clarity, simplicity, and ambiguity. Table 1 shows the scale information for experts.

**Table 1:** Scale information for experts

**Relevance**	**Clarity**
1. Item not relevant to the domain	1. Item is not clear to the domain
2. Item is somewhat relevant to the domain	2. Item is somewhat clear to the domain
3. Item is relevant to the domain	3. Item is clear to the domain
4. Item is very relevant to the domain	4. Item is very clear to the domain
**Simplicity**	**Ambiguity**
1. Item is not simple	1. Doubtful
2. Item need some revision	2. Item need some revision
3. Simple but need minor revision	3. No doubt but need some revision
4. Very simple	4. Meaning is clear

Two-content validity indexes (CVI) had been computed in this study: content validity of the individual item (I-CVI) and content validity of the overall scale (S-CVI/Ave). Content validity of the individual item had been calculated as the total number of experts’ ratings on a scale of three or four divided by the total number of experts. While the content validity of the overall scale had been computed by taking the total proportion and then dividing them by the total components in the instrument. S-CVI (Average) was calculated by using average I-CVI. 0.8 must be the minimal value for S-CVI as recommended by previous studies [27, 28]. The content validity process of the instrument had been completed by dropping the questions that have a minimal agreement and the amendments of the questions depend on the expert’s recommendations.

### Data analysis

SPSS version 26 has been used to examine the data. Factor analysis, Cronbach’s Alpha, and test–retest reliability (Intraclass correlation coefficient, Kappa coefficient) after an interval of two weeks had been used to analyze the data for this study. The best duration between tests differs depending on the instrument being measured, the stability of the instrument by the time, and the target population. Previous research reported that an interval of two weeks is the most recommended [29]. Repeated measures ANOVA with Bonferroni adjustment was used to look for possible factor effects (age, gender) across the time points (test and retest). ANOVA was conducted for the total score of all domains. The SEM was calculated by first creating a variable for the difference between the score obtained during the first and the second administration (test score—retest score = Difference). Next, we calculated the standard deviation of the Difference in our sample (SDdifference) and divided the obtained value by the square root of 2 [30, 31]. The SDCind was calculated with the formula [SDCind = 1.96 x √2 × SEM], and the SDCgroup was calculated by dividing the SDCind by the square root of the number of subjects in the sample [30].

### Factor analysis

We ran the principal factor analysis to check the construct validity. We started with the Kaiser–Meyer–Olkin test and Bartlett’s Test of Sphericity to check the sampling sufficiency for the factor analysis. Factor analysis was then conducted using principal component analysis extraction and the rotation was run by an Equamax method with the option of suppressing small coefficients (absolute value below 0.30). The minimal required value for factor analysis for this study was 0.4 [32].

### Reliability

The process of verifying whether the respondent’s answer had consistency is known as reliability [33]. We employed different ways of analysis to evaluate the reliability in this study: Cronbach’s Alpha and test–retest reliability (Intraclass correlation coefficient (ICC), Kappa coefficient) after an interval of two weeks. Cronbach’s Alpha is often implemented as a reliability test [34]. It has been suggested that 0.7 and above Cronbach’s Alpha indicates that the questionnaire has good reliability [33]. An ICC of 0.4—0.75 indicates acceptable reliability, and a value of more than 0.75 indicates excellent reliability [35]. Linear weighted kappa was used for ordinal items while Cohen’s kappa was used for categorical items. A kappa value of 0.60-0.79 indicated moderate agreement while a value of 0.80—0.90 indicated strong strength of agreement between test and retest [36]. Repeated measures analysis of variance (ANOVA) was used to assess the differences between the test and retest with associated factors (age, gender). Additionally, the standard error measurement (SEM) and minimal detectable change (MDC) for each domain were measured.

## Results

A total of 188 high school students which comprised 57 male (30.3%) and 131 female (69.7%) students participated in this study. Most of the students were Malay (*n* = 133, 70.7%). Yaghmaei [37] proposed that a CVI of more than 0.75 is considered acceptable. As the CVI for all items of our questionnaire was over 0.75, so the questionnaire had acceptable content validity. All the components that had a minimum agreement in terms of their relevance were eliminated because they will elicit similar responses to other components in the questionnaire. Item 7 (slimming tea and other drugs could be used in place of exercise to achieve the same effects) did not reach the required level of CVI in terms of its relevance, clarity, simplicity, and ambiguity so it was removed from the instrument. Table 2 shows the result of content validity.

**Table 2:** Result of content validity

Item No	Relevance	Clarity	Simplicity	Ambiguity	Interpretation
**1**	1	0.83	1	0.83	Acceptable
**2**	1	0.83	1	0.66	Need revision
**3**	1	0.83	0.66	0.66	Need revision
**4**	0.83	0.66	0.66	0.5	Need revision
**5**	1	0.83	1	0.83	Acceptable
**6**	1	0.83	0.83	0.83	Acceptable
**7**	0.66	0.66	0.5	0.5	unacceptable
**8**	1	0.83	0.83	0.83	Acceptable
**9**	1	0.83	0.83	0.83	Acceptable
**10**	1	0.83	0.83	0.83	Acceptable
**11**	0.83	0.83	1	0.83	Acceptable
**12**	1	1	1	1	Acceptable
**13**	1	0.83	0.66	0.66	Need revision
**14**	1	1	1	1	Acceptable
**15**	0.83	1	0.83	0.83	Acceptable
**16**	1	1	1	1	Acceptable
**17**	0.83	0.83	0.83	0.83	Acceptable
**18**	0.83	0.83	0.83	0.83	Acceptable
**19**	1	1	1	1	Acceptable
**20**	1	0.83	0.83	0.83	Acceptable
**21**	1	1	1	0.83	Acceptable
**22**	1	1	1	0.83	Acceptable
**23**	1	1	1	0.83	Acceptable
**24**	1	1	1	1	Acceptable
**25**	1	0.83	0.83	0.83	Acceptable
**26**	1	0.83	0.83	0.83	Acceptable
**27**	1	1	1	1	Acceptable
**28**	1	1	1	1	Acceptable
**29**	1	1	1	1	Acceptable
**30**	1	1	1	1	Acceptable
**31**	1	1	1	1	Acceptable
**32**	1	1	1	1	Acceptable
**33**	1	1	1	1	Acceptable
**34**	1	1	1	1	Acceptable
**Mean I-CVI**	0.96	0.90	0.90	0.86	
**S-CVI/Ave**	0.96	0.90	0.90	0.86	

Abbreviations: *I-CVI* Content validity of the individual item, *S-CVI/Ave* Content validity of overall scale

Questions 2, 3, 4, and 13 had a CVI of more than 0.75 for their relevance but did not reach the required level in terms of clarity, simplicity, and ambiguity. The experts gave some recommendations for items 3 and 13. Item 3 (Washing and other house chores are enough exercise to maintain good health) “could be ambiguous to the participants of this questionnaire” as the expert commented and he suggests modifying the item to (Household chores such as dishwashing is an exercise good enough to maintain health). Item 13 (I use little pain from previous exercises or being tired as an excuse to keep away from further exercises) “The word (soreness) perhaps replaces with pain” as the expert commented. Some modifications also had been done to this item by another expert. Therefore, the item had been modified to (I use little soreness from previous exercises or being tired as an excuse to keep away yourself from further exercises exercising more). The experts did not give recommendations for items 2 and 4 to improve those items regarding these issues. Thus, questions 2 and 4 were not dropped but kept for probable modification after the pilot study. In the pilot study, ten respondents were asked about clarity, ambiguity, and the simplicity of these questions. Based on students’ opinions, the questions are clear and their words simple and easy to understand. Thus, the questions were not dropped. Table 3 displays the result of the Kaiser–Meyer–Olkin test and Bartlett’s Test of Sphericity.

**Table 3:** Result of Kaiser–Meyer–Olkin test and Bartlett’s Test of the Sphericity

Test	Result
Kaiser–Meyer–Olkin Measure of Sampling Adequacy	0.686
Bartlett's Test of Sphericity Approx. Chi square	2086.129
Degree of freedom	528
Significance	< 0.001*

^*^ *p* < *0.05*

Kaiser–Meyer–Olkin test of sampling suitability for factor analysis resulted in a value of 0.686 while the result of significant Bartlett’s test was (*p* < 0.001), indicating that the sample was adequate to proceed to the factor analysis. Table 4 shows the result of the factor analysis. Three questions were removed from the instrument because those questions had a factor loading lower than 0.4, hence did not contribute to a minimal factor structure as shown in the result of a rotated component matrix. The first draft of the questionnaire has about nine questions in the knowledge scope, 10 questions in the attitude scope, nine questions in the participation scope, and five questions in the scope of the experience. Some items have low factor loading in their initial scope but, have high value in another scope as the result of the rotated component matrix shown. Hence, these questions were not dropped but instead shifted into the appropriate domain as a factor loading result showed.

**Table 4:** Result of factor analysis

Domain & components	Final Item	Factor Loading	ICC value	95% Confidence Interval
Knowledge towards exercise (9 items)	Exercise is necessary to maintain good health	0.724	0.736	0.547—0.846
	Exercise can be aerobic or anaerobic	0.699	0.878	0.791—0.929
	Aerobic exercise include jogging, swimming, biking, running, brisk walking	0.478	0.841	0.727—0.907
	Exercise should be done continually throughout life for good health	0.737	0.792	0.644—0.879
	Regular exercise maintaining a normal blood pressure range	0.677	0.882	0.798—0.931
	Regular exercise increasing and maintaining muscular strength and endurance	0.702	0.890	0.811—0.936
	Regular exercise increasing and maintaining flexibility	0.544	0.875	0.785—0.927
	Doing regular exercise is good for my fitness and health	0.607	0.989	0.980—0.993
	I should exercise regularly for my health	0.512	0.941	0.899—0.966
Attitude towards exercise (7 items)	I feel that I have no time of my own and daily exercises take away my valuable time	0.740	0.962	0.935—0.978
	I feel that exercise takes away most of my energy	0.674	0.991	0.985—0.995
	I use little soreness from previous exercises or being tired as an excuse to keep away yourself from further exercises exercising more	0.671	0.988	0.980—0.993
	I give up on exercising owing to a difficulty of sticking to a schedule	0.670	0.961	0.933—0.977
	Household chores such as dishwashing is an exercise good enough to maintain health	0.505	0.986	0.975—0.992
	I need someone to keep reminding me to exercise	0.501	0.936	0.891—0.963
	Exercise does more harm than good	0.409	0.961	0.934—0.978
Participation in exercise (3 items)	In a typical week, on how many days do you walk for at least 10 min continuously to get to and from places?	-0.504	0.961	0.932—0.977
	How much time do you spend doing vigorous-intensity sports, fitness or recreational activities on a typical day?	-0.516	0.977	0.960—0.987
	How much time do you spend doing moderate-intensity sports, fitness? Or recreational (leisure) activities on a typical day	-0.488	0.988	0.979—0.993
Exergame experiences (4 items)	Did you know what is exergames? Such as Xbox 360 and Nintendo Wii	0.819	0.962	0.935—0.978
	Did you try to play with exergames?	0.779	0.916	0.856—0.951
	Have you seen anyone played exergames?	0.731	0.877	0.789—0.928
	Has anyone suggested you to use exergames?	0.718	0.829	0.706—0.900

Abbreviation:

*ICC* Intraclass correlation coefficient

The Cronbach’s Alpha values for the knowledge domain were higher than 0.70 which is acceptable. The total Cronbach’s Alpha if an item deleted is 0.795 which is less than the overall Cronbach’s Alpha value of 0.797 for the knowledge domain so, no item was deleted. The Cronbach’s Alpha values for the attitude domain were also higher than 0.70 which was reliable. The total Cronbach’s Alpha if the item deleted is 0.743 which is a little higher than the overall Cronbach’s Alpha value of 0.738, However, considering the importance of this item (exercise does more harm than good), Q6 was not deleted from the attitude domain. The Cronbach’s Alpha value for the participation domain was 0.520 after deleting seven items from the domain. However, the Cronbach’s Alpha values for the experiences domain were high (0.77) which is acceptable. The total Cronbach’s Alpha value if an item is deleted for this domain is 0.753 which is less than the overall value of the domain. Hence, no item was deleted. The ICC values for the test–retest reliability after an interval of two weeks for all questions were above 0.7 as shown in Table 5 which is acceptable and indicates good reliability. Weighted Kappa ranges between 0.67–0.95 and 0.86–0.96 for the knowledge and attitude domains, respectively, while Cohen’s kappa for domain 4 (categorical items) ranges between 0.70–0.92 as shown in Tables 6 and 7.

**Table 5:** Summary of the factor analysis and reliability of the final questionnaire

Domains and components	Initial items	Final items	Factor loading	95% CI (Overall ICC value)	SEM	SDC_ind_	SDC_group_
Knowledge	9	9	0.478—0.737	0.7—0.9 (0.547—0.980)	1.47	4.07	0.54
Attitude	10	7	0.409—0.740	0.9 (0.891—0.993)	0.96	2.66	0.35
Practice	9	3	0.488—0.516	0.9 (0.932—0.993)	0.46	1.27	0.17
Experiences	5	4	0.718—0.819	0.8—0.9 (0.706—0.978)	0.34	0.94	0.12

Abbreviation: *ICC* Intraclass correlation coefficient, *SEM* Standard error of measurement, *SDCind* Smallest detectable change for individual subject, *SDCgroup* smallest detectable change for group. CI = Confidence Interval

**Table 6:** Result of weighted kappa for ordinal scale

	**Kappa**	***p*-value**
**Items in knowledge scale**		
Exercise is necessary to maintain good health	0.679	< 0.001
Exercise can be aerobic or anaerobic	0.856	< 0.001
Aerobic exercise include jogging, swimming, biking, running, brisk walking	0.716	< 0.001
Exercise should be done continually throughout life for good health	0.735	< 0.001
Regular exercise maintaining a normal blood pressure range	0.805	< 0.001
Regular exercise increasing and maintaining muscular strength and endurance	0.816	< 0.001
Regular exercise increasing and maintaining flexibility	0.788	< 0.001
Doing regular exercise is good for my fitness and health	0.952	< 0.001
I should exercise regularly for my health	0.854	< 0.001
**Items in the attitude scale**		
I feel that I have no time of my own and daily exercises Take away my valuable time	0.899	< 0.001
I feel that exercise takes away most of my energy	0.961	< 0.001
I use little soreness from previous exercises or being tired as an excuse to keep away yourself from further exercises exercising more	0.948	< 0.001
I give up on exercising owing to a difficulty of sticking to a schedule	0.882	< 0.001
Household chores such as dishwashing is an exercises good enough to maintain health	0.966	< 0.001
I need someone to keep reminding me to exercise	0.862	< 0.001
Exercise does more harm than good	0.929	< 0.001

**Table 7:** Result of Cohen’s kappa for categorical scale

Items in the scale of the experience	Kappa	*p*-value
Did you know what is exergames? Such as Xbox 360 and Nintendo Wii	0.927	< 0.001
Did you try to play with exergames?	0.843	< 0.001
Have you seen anyone played exergames?	0.781	< 0.001
Has anyone suggested you to use exergames?	0.707	< 0.001

The SEM for the different domains of the questionnaire varied between 0.34 for domain 4 and 1.47 points for domain 1. The SDCind for all domains ranges from 0.94 points for domain 4 to 4.07 points for domain 1. The SDCgroup for the questionnaire goes from 0.12 for domain 4 to 0.54 for domain 1. The SEM, SDCind, and SDCgroup values for all domains are presented in Table 5. Repeated measures ANOVA was used to look for possible factor effects (age, gender) across the time points. The result of the data did not reveal significant interactions between gender and age across time. The result of repeated measures ANOVA for the total score of all domains has been shown in Table 8 (*p* > 0.05 for all domains).

**Table 8:** Repeated measure ANOVA of the differences between test and retest with associated factors (age, gender)

	**Effect**	**MS**	**df**	***F* value**	***p*-value**
**Tests of Within Subject Effects**					
Knowledge	Time	0.088	1	0.035	0.853
	Time x Age	0.502	4	0.197	0.938
	Time x Gender	0.051	1	0.020	0.888
	Time x Age x Gender	0.577	4	0.227	0.922
	Error	2.545	41		
Attitude	Time	0.266	1	0.275	0.603
	Time x Age	0.462	4	0.478	0.752
	Time x Gender	0.102	1	0.106	0.747
	Time x Age x Gender	0.625	4	0.647	0.632
	Error	0.967	41		
Practice	Time	0.215	1	0.854	0.361
	Time x Age	0.074	4	0.296	0.879
	Time x Gender	0.215	1	0.854	0.361
	Time x Age x Gender	0.074	4	0.296	0.879
	Error	0.251	41		
Experiences	Time	0.088	1	0.826	0.369
	Time x Age	0.220	4	2.062	0.104
	Time x Gender	0.177	1	1.660	0.205
	Time x Age x Gender	0.242	4	2.267	0.078
	Error	0.107	41		
**Tests of Between-Subject Effects**					
Knowledge	Age	11.326	4	0.708	0.591
	Gender	2.245	1	0.140	0.710
	Age x Gender	7.097	4	0.443	0.777
	Error	16.008	41		
Attitude	Age	27.570	4	0.782	0.544
	Gender	2.044	1	0.058	0.811
	Age x Gender	79.487	4	2.254	0.080
	Error	35.261	41		
Practice	Age	9.445	4	1.856	0.138
	Gender	0.009	1	0.002	0.967
	Age x Gender	6.983	4	1.355	0.268
	Error	5.089	41		
Experiences	Age	3.624	4	0.928	0.457
	Gender	1.389	1	0.356	0.554
	Age x Gender	3.591	4	0.920	0.462
	Error	3.904	41		

Abbreviation: *MS* Mean square, *df* Degrees of freedom

## Discussion

KAP survey is widely used to measure the level of knowledge, attitude, and practice towards exercise. However, to the best of our knowledge, there is no questionnaire assessing the KAP towards exergames, so we modified an existing questionnaire adopted from a previous study [24] with an additional section to assess exergames experiences. The questionnaire of this research consists of 4 categories: knowledge, attitude, and practice towards exercise that can identify information that is commonly known and attitudes that are commonly held and practices related to exercise. The fourth section consists of four questions that focus on exergames experiences. The sample size had to be considered to define the suitability of the data for factor analysis. Hair et al. [38] recommended that sample sizes have to be at least a hundred or more. The sample size calculation for factor analysis primarily depends totally on the total components of the instrument. It has been recommended to be with a ratio of 5:1 (5 respondents for each item) [39]. Therefore, the sample size for running factor analysis in our study should not be below 170 students (34 questions*5). Depending on those suggestions about defining the appropriateness of sample size for factor analysis, thus a sample size of 188 students was included in this study. On the other hand, Hertzog et al. [40] argued that a sample size of 40 per group might be sufficient for test–retest reliability. Hence, the total sample size needed for this study was 55.

The first draft of the questionnaires consists of around 9 questions in the knowledge part, 10 questions in the attitude part, 9 questions in the participation part, and 5 items in the awareness part. Two items in the participation domain and one item in the exergames domain had been removed due to those questions did not meet the minimal factor loading of 0.4. In the knowledge domain, some items show low factor loading but have high factor loading in the attitude domain as the rotated component matrix shows (factor loading = 0.409, 0.505). Hence, these questions were not dropped but shifted into the appropriate domain. Some items in attitude domains show low factor loading but have high factor loading in the knowledge domain (factor loading = 0.512, 0.607, 0.737). As a result, these items were not eliminated but shifted into the knowledge part. The final version of the questionnaire contained nine questions in the knowledge part. Like these results, some items in the attitude domain have low factor loading in their initial domain but have a high factor loading in the participation domain (factor loading = 0.410, 0.508, 0.597). Hence, these items were not dropped but shifted into the participation domain as the rotated component matrix shown. The final version of the questionnaire contained seven items in the attitude domain and 10 items in the participation domain as the rotated component matrix is shown. The exergames domain remained with no change (four items).

Repeated measures ANOVA did not show any significant differences between test and re-test for all questionnaire domains. The result of the data suggested that the time effect is not likely. Furthermore, the result did not reveal significant interactions between gender and age in the knowledge, attitude, practice, and awareness across time.

The Cronbach’s Alpha values of knowledge, attitude, and experiences domains were 0.797, 0.738, and 0.779 respectively. The ICC value for these domains were 0.7 -0.9, 0.9, and 0.8—0.9 respectively, which indicate that it is acceptable and have good reliability. The weighted kappa value for the knowledge and attitude domain ranges between 0.67—0.96 while Cohen’s kappa for the experience’s domain was between 0.70—0.92 which reflects moderate to almost strong strength of agreement between test and retest. The results of SEM for the 4 domains of the questionnaire showed low SEM and SDC values. The largest SME value was 1.47 for the knowledge domain. However, all SEM values were relatively stable over the domains.

The Cronbach’s Alpha values of the participation domain were low and then increased to 0.520 after deleting seven items. However, a 0.5 to 0.75 value count is generally accepted and indicates a moderately reliable scale [41]. The ICC value of this domain ranges between 0.7–0.9 which indicates good reliability. The decision to drop the seven questioners was because the remaining three questions in this domain can measure the participation in different types of exercise based on the opinion of an exercise scientist. The first question: In a typical week, how many days do you walk for at least 10 min continuously to get to and from places? This question measures the participation level in low-intensity exercise. The second question: How much time do you spend doing moderate-intensity sports, fitness, or recreational (leisure) activities on a typical day? This question measures the participation level in moderate intensity exercise while the third question measures the participation level in vigorous-intensity exercise; How much time do you spend doing vigorous-intensity sports, fitness, or recreational activities on a typical day?

## Limitations and future research direction

There were a few limitations of this study. Firstly, the low response rate, which was expected for an online survey. However, the study was able to achieve an adequate sample size to ensure the validity and reliability of the questionnaire despite the low response rate. Another limitation was language bias. As the questionnaire was written in English. So, the participants who understand English only can participate in this study. Future studies may focus on item improvements such as including Likert-type questions that allow obtaining more information from the participants. In addition, a translated version of the survey may be more appropriate to be used in future study to facilitate the data collection process.

## Conclusion

Our questionnaire can be used to assess knowledge, attitude, and practice regarding exercise and exergames experiences among high school students as it has acceptable validity and reliability. More research with different populations is needed to determine whether our findings are sample-specific or more universal.

## Supplementary Information

**Additional file 1.** 

## Data Availability

All data generated or analyzed during this study are included in this published article [and its supplementary information files].
